# An FMCW Radar for Localization and Vital Signs Measurement for Different Chest Orientations

**DOI:** 10.3390/s20123489

**Published:** 2020-06-20

**Authors:** Giulia Sacco, Emanuele Piuzzi, Erika Pittella, Stefano Pisa

**Affiliations:** 1 Department of Information Engineering, Electronics and Telecommunications, Sapienza University of Rome, 00184 Rome, Italy; emanuele.piuzzi@uniroma1.it (E.P.); stefano.pisa@uniroma1.it (S.P.); 2 Department of Legal and Economic Sciences, Pegaso University, 00186 Rome, Italy; erika.pittella@unipegaso.it

**Keywords:** FMCW radar, vital signs, AAL

## Abstract

This work tests the ability of a frequency-modulated continuous wave (FMCW) radar to measure the respiratory rate and the heartbeat of a subject in challenging indoor scenarios. To simulate a realistic configuration for ambient assisted living (AAL) applications, in which the thorax orientation towards the antenna is typically unknown, four different scenarios were considered. Measurements were performed on five volunteers positioned with the chest, left, back, and right side facing the antenna, respectively. The 5.8 GHz radar and the antennas used for the measurements were suitably designed for the considered application. To obtain a low cost and compact system, series-fed arrays were preferred over other antenna topologies. The geometry of the patches was opportunely shaped to reduce the side lobe level (SLL) and increase the bandwidth, thus ensuring good system performances. In all scenarios, the vital signs extracted from the radar signal were compared with the ones collected by a photoplethysmograph and a respiratory belt, used as references. A statistical analysis of the measured data on the different subjects and orientations was performed, showing that the radar was able to measure with high accuracy both the respiratory rate and the heartbeat in all considered configurations.

## 1. Introduction

With the population aging worldwide, new solutions for non-invasive health monitoring are required. Radar systems have been proposed as a promising technology for vital signs’ monitoring in ambient assisted living (AAL) applications [1,2,3,4]. Their major advantage is that, without the need for any cable or electrode, it is possible, at first, to locate the patient inside the room and, then, to measure his/her respiratory rate and heartbeat. In [5,6,7,8,9,10,11,12,13,14], impulse radars were presented as a valid solution for the vital signs’ estimation. However, because of their broadband nature, they are power consuming and require complex architectures and high speed analog-to-digital converters (ADC). Thanks to their lower power consumption, continuous wave (CW) radars [15,16,17,18,19,20,21,22,23,24,25,26] have been proposed as a valid alternative to impulse radars. This family of sensors can accurately measure the chest movements connected with the heart and the respiratory activity, but they fail to provide any information about the patient’s distance. To overcome this limitation, hybrid systems integrating both a CW and a frequency-modulated continuous wave (FMCW) radar were considered in [27,28]. In this case, the CW radar is used for vital signs’ estimation, and the FMCW one is used for patient localization. Even if all the required information can be accurately retrieved with this configuration, a complex hardware architecture and two different signal processing chains are needed. In [29], it was shown that, with a single FMCW radar, both the information about the vital signs and the position could be concurrently measured. In particular, vital signs could be obtained from the analysis of the phase variations associated with the target position. Another advantage of FMCW radars, compared to CW ones, is that they are less affected by clutter and multipath. In fact, with CW radars, the received signal is degraded by all the noise generated by the surrounding environment, while in FMCW radars, the target can be spatially separated from the disturbing objects located in different range bins, hence guaranteeing an intrinsic filtering. FMCW radar systems have already been proposed in the past for multiple applications related to vital signs’ monitoring and AAL applications. In [30,31], this kind of sensor was proven able to detect fall events, while in [32], an FMCW radar operating at 24 GHz was positioned on a wheelchair for the vital signs’ monitoring. In [33], the tracking ability of this kind of system together with the ability to retrieve the respiratory and heart rate in the presence of the target’s small movements were demonstrated.

In typical AAL applications, the vital signs’ monitoring should be performed continuously and during the daily activities of the patient, meaning that the radar should retrieve the respiratory and heart rate independently of the chest orientation towards the radar antenna. However, if the patient’s chest is not facing the antenna, vital signs’ measurements are challenging. Indeed, even though the entire rib cage expands, due to the respiratory and heart activity, the excursions are stronger in the front side than in the lateral and back side.

In this work, we propose a detailed analysis of an FMCW radar’s ability to retrieve the respiratory and heart rate independently of the patient orientation towards the antenna. The 5.8 GHz radar system used for the measurement was designed specifically for the proposed application. In particular, a new geometry was used in the transmitting and receiving antennas, in the form of two identical series-fed arrays with a high gain, low side lobe level, and wide band. This kind of antenna is traditionally used in radar systems; however, it suffers from a reduced bandwidth and a high side lobe level. With the new shape we propose for the antenna patches, the complete 5.8 GHz Industrial, Scientific, and Medical (ISM) band is covered, with a side lobe level of about −20 dB. The antenna high gain is a fundamental aspect of this system, because it can be leveraged to spatially filter out multi-path and clutter and obtain accurate measurements even when the environment or the subject’s orientation is unfavorable. To confirm the broad validity of the results, we performed the vital signs’ measurements in a realistic and challenging indoor setting, and we measured five subjects (three males and two females) aged between 25 and 63 years, without any known underlying cardiac or respiratory pathology. The chosen subjects showed different values of heart rate, ranging from 60 to 100 beats per minute (BPM), thus allowing testing the radar for different physiological conditions. In particular, four different configurations were considered: the measured subjects were positioned with the chest, the left side, the back, and the right side facing the antenna. A detailed statistical analysis of the results was performed to compare the radar detection ability to the one of the gold standards and also to show the proposed system performance as compared to other solutions available in the literature.

In Section 2, the displacements of the chest due to the respiratory and heart activities are described to better clarify which quantities are really measured by the FMCW radar and how they can vary according to the position of the thorax. In Section 3, the ad hoc designed radar and radiating elements are described. In Section 4, the signal processing chain is presented, and in Section 5, the measurement results are shown. In Section 6, conclusions are drawn.

## 2. Problem Geometry

**Figure 1:**
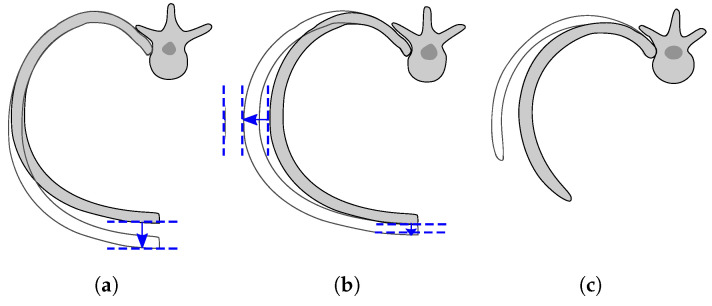
Chest ribs’ movements during respiration: (**a**) pump-handle motion of the upper ribs, (**b**) bucket-handle motion of the lower ribs, and (**c**) caliper motion of the lowest ribs.

Radar systems used for AAL applications must concurrently measure two pieces of information: (i) the patient’s position inside the environment and (ii) the small movements of the chest caused by his/her respiratory activity and heartbeat. Regarding the respiratory activity, three different movements of the rib cage can be distinguished: the “pump-handle” motion, the “bucket-handle” motion, and the “caliper” motion [34,35]. The dominant movement for the upper ribs is the pump-handle (Figure 1a), responsible for the variation of the chest dimension in the anteroposterior direction. The lower ribs instead, due to their different connection to the spine, maintain an approximately constant position in the front side, mainly modifying the lateral dimensions of the thorax (Figure 1b).

The lowest ribs, also known as floating ribs, are not connected to the sternum and during the respiratory activity tend to flare open and backward, rotating in a caliper motion around the connection they have with the spine (Figure 1c). Measurements of the chest displacement performed by [36] indicated that the maximum excursion of the thorax was 4.33 mm on the front side, 1.21 mm on the lateral side, and 1.71 mm on the dorsal side. The heart, instead, causes a maximal chest displacement when the left ventricle hits the chest wall, at the fourth and fifth intercostal space [35]. In [37], the maximum registered displacement induced by the heart during the QRS was about 0.6 mm. The variation of the displacement entities induced by the respiratory and heart activities in the different areas of the thorax makes the vital sign measurements in typical AAL applications challenging, in which the thorax orientation with respect to the antenna is unknown. This requires an experimental validation to assess the ability of an FMCW radar to measure the respiratory and heart frequency if the target chest does not face the antenna.

## 3. System Overview

### 3.1. Radar Architecture

**Figure 2:**
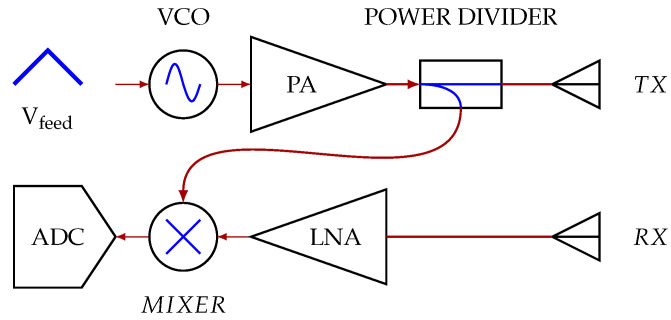
Block diagram of the designed radar. VCO, voltage controlled oscillator; PA, power amplifier; LNA, low noise amplifier.

**Figure 3:**
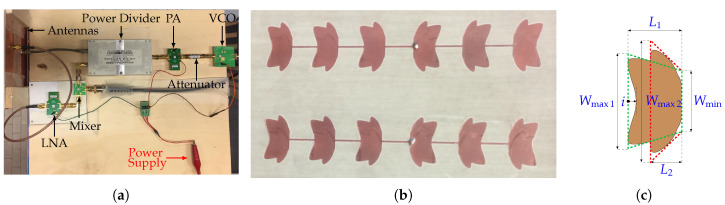
Photo of (**a**) the radar system, (**b**) the antennas, and (**c**) the geometry of the patch.

The designed system is a single-input single-output (SISO) FMCW radar, working in the 5.8 GHz ISM band. The radar was designed in this band since, at these frequencies, the free space wavelength is comparable with the chest excursions during the respiration, thus facilitating the vital signs’ extraction. A block diagram of the system is represented in Figure 2, and the photos of the realized radar and antennas are reported in Figure 3.

The transmitting chain of the radar is composed of a voltage controlled oscillator (VCO), with an output power of 5 dBm, a power amplifier (PA), a power divider, and the transmitting antenna. An attenuator was added between the VCO and the amplifier to reduce the input power to this component and to make it work in the best conditions. For the VCO, a triangularly-shaped feeding signal was preferred to a ramp since it did not present strong discontinuities, which could adversely affect the measured signals with the introduction of unwanted harmonic components. The feeding signal $V_{\mathrm{feed}}$ has a fundamental frequency $f_{T}=300\mathrm{Hz}$ and a duty cycle of 10%. The receiving chain includs, instead, the receiving antenna, a low noise amplifier (LNA), a mixer, and an ADC. To reduce the costs and the dimensions of the system, discrete components were preferred. In particular, the chosen components were HMC587LC4B [38] for the VCO, HMC392ALC4 [39] for both the PA and the LNA, ZN2PD2-63-S+ for the power divider [40], and HMC557A [41] for the mixer. The transmission and the reception of the signal were controlled and synchronized by the NI DAQ USB 6361 [42]. Considering that the triangular signal produced by the NI DAQ USB 6361 stayed constant in time, we could assume that also the chirps generated by the VCO were essentially identical.

### 3.2. Antenna Design

**Figure 4:**
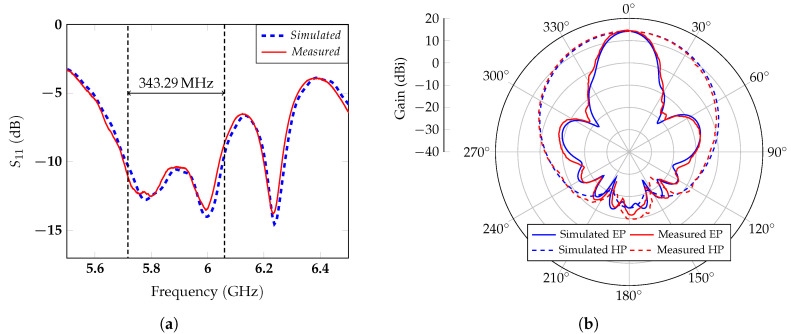
Simulated and measured (**a**) reflection coefficient and (**b**) radiation pattern at 5.8 GHz in the Eand Hplanes (EP and HP).

The antennas were specifically designed for the proposed application [43]. The receiving and transmitting antennas are two identical series-fed arrays composed of six patches. The shape of the patch (see Figure 3c) was suitably modified to increase the bandwidth of this antenna topology, which is typically narrow. Since for FMCW radars, the range resolution is inversely proportional to the bandwidth [44], it was crucial to design an antenna with a band equal to or larger than the one available in the ISM frequency range used. The bandwidth increase was achieved through a dual band structure, given by the superposition of two tapered patches. These patches were obtained by spline interpolation within the red and the green dashed polygons in Figure 3c. The two resonating frequencies depend on the lengths $L_{1}$ and $L_{2}$ in Figure 3c. To reduce the side lobe level (SLL), an alternative technique to the amplitude tapering was applied. The curvature degree of the upper edge of the patch, controlled by the indent parameter *i* in Figure 3c, was used to modulate the amount of power transferred from one patch to the following ones. In particular, enlarging the indent increases the power radiated by the patch and reduces the transmitted one. Feeding the antenna from one of the two central patches (see Figure 3) and choosing the appropriate value of *i*, a superficial current distribution ensuring a higher radiation from the central patches than from the most external ones could be guaranteed, thus allowing a fine regulation of the SLL. Furthermore, it is worth noting that, contrary to what happens for the amplitude modulation methods, the presented SLL control does not require a variation of the patch shape along the array. With the proposed design, the antenna has a fractional bandwidth of 5.92%, which is more than the double the 2.6% required and wider than the ones proposed in the literature for an equivalent antenna topology [45,46]. The antenna has a measured gain of 14.3 dBi at the central frequency of 5.8 GHz. Having a high directivity helps concentrate the antenna’s main beam on the patient’s chest, thus minimizing the influence of the clutter coming from the surroundings. Two prototypes of the designed antenna were realized with a computer numerical control milling machine (see Figure 3b). The reflection coefficient and the radiation pattern of the antenna in both the E plane (EP) and in the H plane (HP) were measured. The comparison between the simulations and measurements is reported in Figure 4.

At 5.8 GHz, the measured SLL was −21.6 dB, and the main lobe angular width 20 ${}^{\circ }$, in excellent agreement with the simulation results.

## 4. Signal Processing

**Figure 5:**
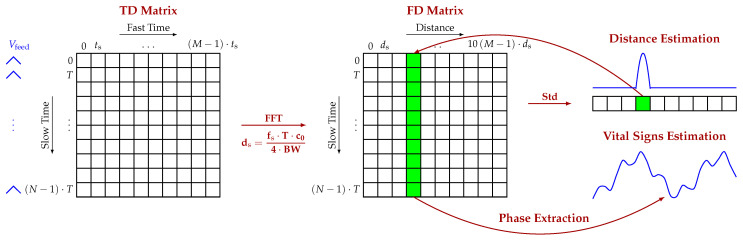
Signal processing chain: TD matrix, FD matrix resulting from the fast Fourier transform (FFT), standard deviation (std) array for the distance estimation, and phase extraction for the vital signs’ retrieval.

The signal measured by the radar was collected with an NI DAQ USB 6361 with a sampling frequency $f_{s}=1/t_{s}=50\mathrm{kHz}$. Each acquisition lasted $T_{a}=30\;s$, a sufficient amount of time to accurately estimate the patient’s vital signs. The samples collected during the acquisition were arranged into a 2D matrix (Time Domain (TD) matrix in Figure 5) with *N* rows and *M* columns.

The number of rows *N* corresponds to the number of triangular signals sent during the acquisition time ($N=T_{a}/T$), while the number of columns is equal to the number of samples collected during one period of the signal $V_{\mathrm{feed}}$. To extract the range information, an FFT in the fast time (i.e., along the rows) of the TD matrix was computed and stored in the Frequency Domain (FD) matrix. Since in the 5.8 GHz ISM band, the bandwidth is only 150 MHz, the range resolution of the FMCW radar is quite poor, thus limiting the possibility to estimate the target position correctly.

**Figure 6:**
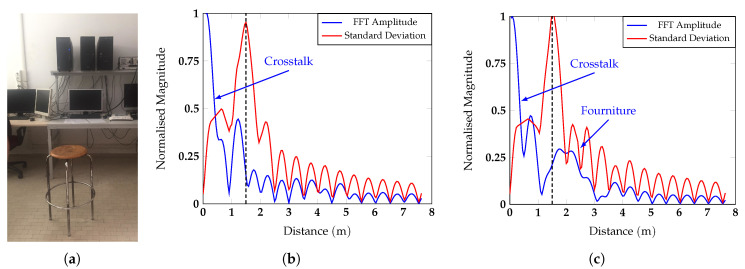
(**a**) Photo of the measurement environment and the effect of the standard deviation on the radar signal (**b**) when the subject had the chest facing the antenna and (**c**) when the subject had the side towards the antenna.

To overcome this limit, each row of the matrix was padded with a number of zeros equal to nine times the length of the row *M*. The standard deviation of the FD matrix in the slow time (i.e., along the columns) was then computed. This second operation is crucial in typical indoor environments in which the measured subject is not the only target. The radar signal could be scattered by the elements of furniture, which depending on their material and dimension, could introduce reflections much stronger than the ones of a human subject. Furthermore, the radar crosstalk could also negatively impact the measurements, producing a peak on the FFT signal that is stronger than that of the target. With this operation, both contributions can be reduced, and the subject can be clearly be distinguished from static objects thanks to the movements of his/her chest. The ability to distinguish the target from the surrounding clutter correctly was further enhanced by the high directivity of the designed antenna. In fact, if the angular width of the main beam is small, the radiation can be concentrated on the patient’s chest, thus helping to filter the clutter spatially. In the room where the measurements were taken, the subjects were seated on a stool positioned 1.5 m away from the radar, and a desk with some computers was positioned 1 m behind the target. Figure 6 reports two examples of the target position estimation based on the amplitude of the FFT and on the standard deviation.

In particular, Figure 6b represents the case in which the subject’s chest was facing the antenna, while Figure 6c the case in which the patient had the left side of the body towards the antenna. The blue curve in Figure 6 is the FFT amplitude of the radar signal collected during one period of the feeding triangular signal $V_{\mathrm{feed}}$ and padded, while the red curve is the standard deviation of the complex FFT of the TD matrix. In Figure 6b, thanks to the high directivity of the antenna, the main beam is concentrated on the patient’s chest, and the furniture positioned 1 m away from the subject’s back does not appear in the spectrum. In this case, the only unwanted contribution was given by the radar crosstalk and was eliminated with the standard deviation. In the orientation of Figure 6c, the subject appears thinner compared to the dimensions of the antenna main beam. For this second scenario, both contributions were eliminated by the standard deviation. Once the range bin corresponding to the target position (green sample of the std array in Figure 5) was defined, the phase of the data column corresponding to the target position (green column of the FD matrix in Figure 5) could be extracted [29]. From the spectrum of the unwrapped phase, the frequencies corresponding to the heartbeat and the respiratory rate were identified.

## 5. Measurement Results

**Figure 7:**
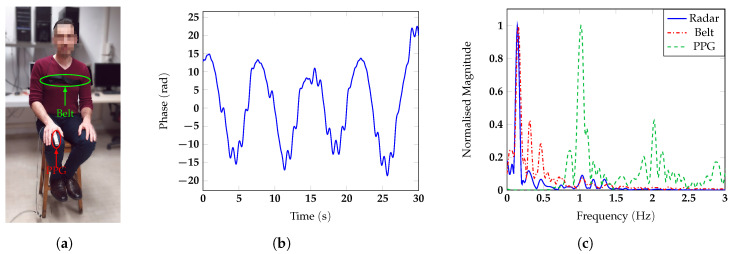
(**a**) Photo of the experimental setup in which the chest of the subject is facing the antenna; (**b**) vital signs extracted by the radar in the time domain and (**c**).

**Figure 8:**
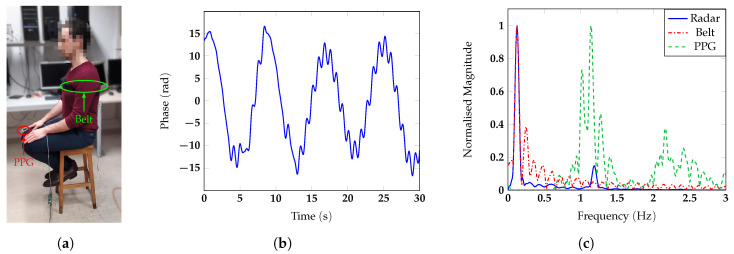
(**a**) Photo of the experimental setup in which the left side of the subject is facing the antenna; (**b**) vital signs extracted by the radar in the time domain and (**c**) spectrum.

**Figure 9:**
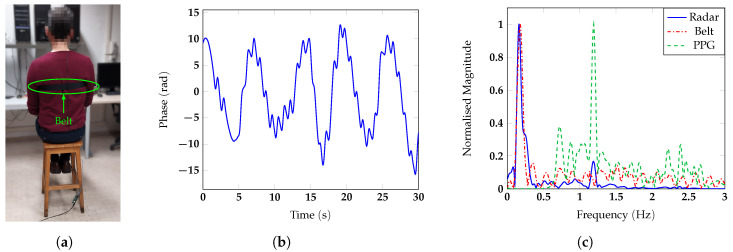
(**a**) Photo of the experimental setup in which the back of the subject is facing the antenna; (**b**) vital signs extracted by the radar in the time domain and (**c**) spectrum.

**Figure 10:**
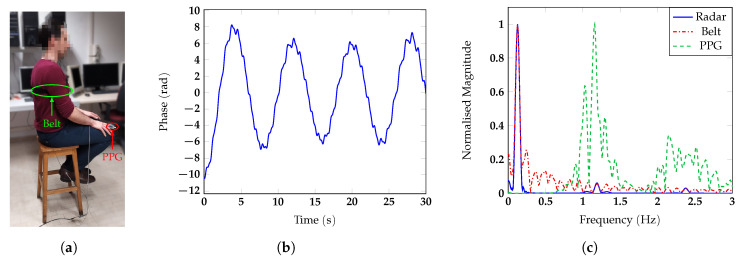
(**a**) Photo of the experimental setup in which the right side of the subject is facing the antenna; (**b**) vital signs extracted by the radar in the time domain and (**c**) spectrum.

To verify the ability of the radar to measure the vital signs independently of the patient chest orientation towards the antenna, four scenarios were considered. Measurements were performed on five subjects, three males and two females, aged from 25 to 63 and without any known underlying cardiac or respiratory pathology. The measured subjects were positioned in front of the radar with the chest, left side, back, and right side facing the antenna. In all the configurations, the target was 1.5 m away from the antenna, and he/she was invited to breath normally. To test the measurement accuracy, a respiratory belt and a finger photoplethysmogram (PPG) were used as references for respiratory and heart rate, respectively. The photos of the different configurations with one of the subjects are reported in Figure 7a, Figure 8a, Figure 9a and Figure 10a. The two reference sensors positions are indicated with green and red arrows, except for Figure 9a, in which the PPG is covered by the body of the subject and only the respiratory sensor can be seen.

**Figure 11:**
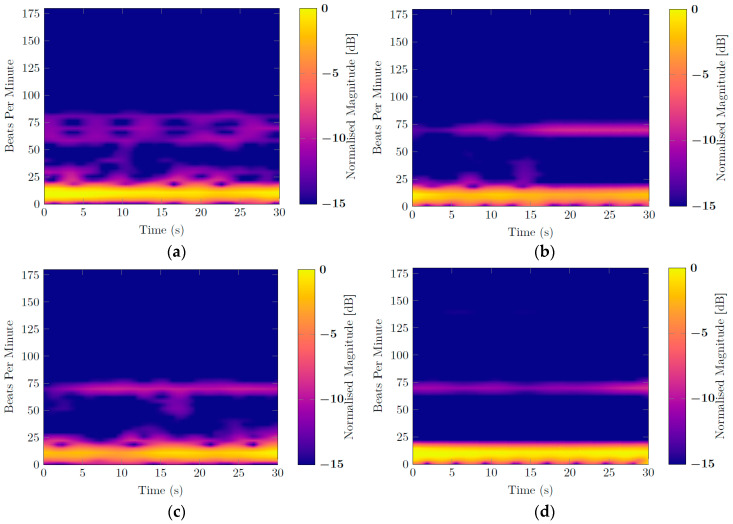
Spectrogram over 30 s of the signal measured by the radar (**a**) with the chest of the subject (**b**) the left side, (**c**) the back, and (**d**) the right side facing the antenna.

Figure 7, Figure 8, Figure 9 and Figure 10 report for one of the subjects, taken as an example, the unwrapped phase extracted at the frequency bin corresponding to the target position (time signal) and the normalized amplitude of the corresponding spectrum for the different configurations. For all the subject positions, the acquisition duration was 30 s, an amount of time sufficiently long to allow for the correct estimation of the vital signs. Two harmonic components could be clearly identified for all the configurations. The accuracy of the measurements was then confirmed by the spectral analysis in which the radar signals were compared with the corresponding references. In all scenarios, the radar signal spectrum showed two peaks emerging distinctly around the frequencies 0.1 Hz and 1 Hz, corresponding to the respiratory and the heart rate, respectively. The positions of these frequency peaks measured by the radar were in good agreement with the ones extracted by the respective references. Figure 11 reports, for the same subject, the spectrograms of the radar signals collected in the four scenarios and computed for the acquisition time of 30 s. For the sake of simplicity in this paper, we used the unit BPM both for the respiratory and heart rate.

The respiratory and heart rate clearly emerged during the whole measurement for all the considered configurations. For the scenario in which the target had the chest facing the antenna, two other harmonic components appeared around the one of the heart, but having a lower amplitude, did not alter the measurement results. In all the configurations, except for the one in which the right side of the body was positioned towards the radar, some additional small harmonics, with an intensity inferior to the one of the heart, appeared in the spectrogram, but they still did not compromise the recognition of the respiratory and heart rates. For all the scenarios, during the measured time, the respiratory frequency and the heartbeat could be successfully retrieved, and no relevant performance loss was noticed for the positions in which the thorax of the measured subject was not facing the antenna. This indicated that the FMCW radar was a good candidate for vital signs’ measurements in challenging AAL applications.

### Statistical Analysis

**Figure 12:**
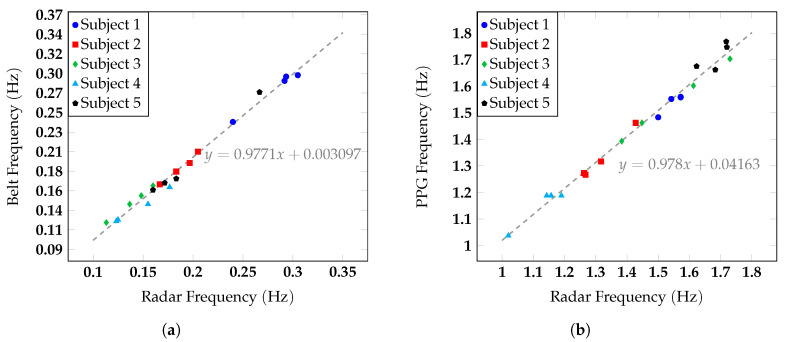
Regression of (**a**) the respiratory rate measured with the respiratory belt and the radar and (**b**) the heart rate measured with the PPG and the radar.

The data collected on the five subjects were then analyzed statistically to characterize the radar performance and to assess if this kind of system could be properly used in typical AAL applications. The data for all the subjects were measured for the four configurations, with an acquisition time of 30 s. The regression of the data measured for both the respiratory and the heart rate is shown in Figure 12a,b.

**Figure 13:**
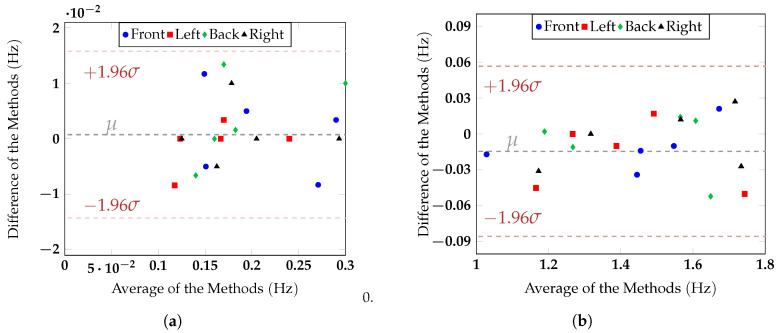
Bland–Altman comparison (**a**) of the respiration rate measured with the respiratory belt and the radar and (**b**) of the heart rate measured with the PPG and the radar. The grey line in the figures refers to the mean (μ), while the red lines define the limits of the confidence interval (μ±1.96σ).

Different symbols were used to distinguish the contributions of the five subjects. The breathing rate of the subjects varied from 0.11 Hz to 0.31 Hz, corresponding to 6.60 BPM and 18.6 BPM, respectively, while the hearth rate ranged from 1.02 Hz to 1.73 Hz, corresponding to 61.2 BPM and 103.8 BPM. Thanks to the strong variations in respiratory and heart rates, it was possible to test the ability of the radar to retrieve vital signs independently of the chest orientation, inside a wide range of values that covered most of the typical average values of these quantities. The regression lines for the respiratory and the heart rate have a slope almost equal to one and more specifically corresponding to 0.9771 and 0.978, for the breath and the heart activity, respectively, thus indicating an excellent agreement between radar and reference measurements. Data dispersion was slightly higher for the heart rate than for the respiration rate, especially for Subject 5. This could be explained by the fact that chest movements induced by the heart beat were lower than the ones produced by the respiration. In Figure 13a,b, the Bland–Altman analysis method was applied to the data.

**Figure 14:**
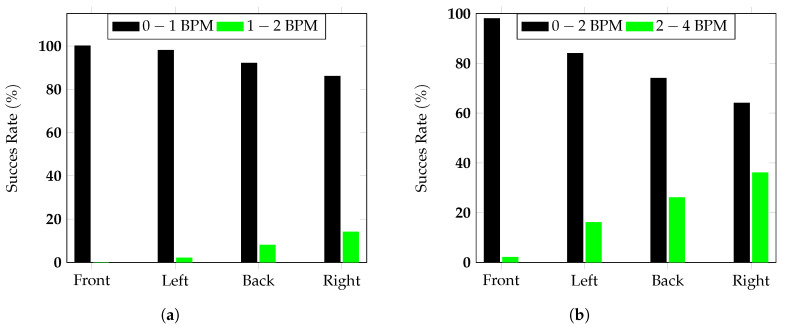
Success rate of the radar measurements (**a**) for the respiration rate and (**b**) for the heart rate.

This technique is normally used to compare two measurement methods, plotting the difference between the methods against the average of the two measurements. In this case, the data were sorted according to the orientations and not to the different subjects, to verify if a significant difference in the measurement error could be noticed for one or more of the considered configurations. Supposing a Gaussian distribution of the data, the 95% confidence upper and lower limits, plotted in Figure 13a,b, were computed as 1.96σ, where σ was the standard deviation. For all the considered configurations, the radar measurement results for the respiration and the heart rate were inside the confidence interval limits defined by the red lines in Figure 13a,b, thus proving the agreement between the two methods. Furthermore, it is worth noticing that for the measured subjects considering an acquisition time of 30 s, the maximum registered error was 0.8 BPM for the respiration rate and 3.1 BPM for the heart rate. The signals were then processed considering sliding windows of 20 s, with overlaps of 19 s. Figure 14 shows the success rate of the measurements, defined as the time the respiration and heart rate measured by the target stayed lower than a specified value of BPM.

For the measured subjects, independently of the orientation, the respiration rate error stayed under 2 BPM in 100% of the measurements and 100%, 98%, 92%, and 86% under 1 BPM when the chest, the left side, the back, and the right were towards the antenna, respectively. For the hearth rate, all the measurement errors stayed under 4 BPM and under 2 BPM for 98%, 84%, 74%, and 64% for the front, left, back, and right orientations, respectively. Finally, for one of the subjects, measurements were repeated at different distances (1 m, 2 m, and 3 m) to test the radar behaviour in different signal-to-noise ratio conditions. For all the considered distances, the error in the respiration rate was less than 1 BPM and the one on the heart rate less than 3 BPM, thus showing that with the considered configuration, the radar was able to estimate the vital parameters for typical distances for AAL applications correctly. The results we obtained are compatible with or outperformed comparable systems presented in the literature. For instance, the 24 GHz FMCW system for heart rate monitoring presented in [47] reported an error of up to 5 BPM for a distance between 160 cm and 300 cm in their frontal heart rate measurements, which compared favourably with our results of less than 4 BPM error obtained in our experiments for up to 3 m. For the FMCW system introduced in [33], in a frontal measurement scenario in which the subject was seated on a sofa with sound absorbing back panels at 2.6 m, the measurement error remained lower than 6 BPM for 99.5% and 90% of the time for the respiration and the heart rate, respectively. In [48], an FMCW radar, working in the X-band, was used to measure the vital signs of six people lying on a bed with the radar positioned on the ceiling at about 2.5 m from the target. The data were processed with different algorithms, and considering sliding windows of 20 s, the error between the gold standard and the heart rate measured by the radar did not exceed 5% for 50.5% of the time in the best case. Contrary to the analysis performed in this paper, no distinction was made in terms of the subject orientation for the error calculation. However, according to the data collected in this work, an error of 5% corresponds to 3.06 BPM for the lowest value of measured heart rate (61.2 BPM) and to 5.19 BPM for the highest one ( 103.8 BPM). It is worth noticing that, for the right-side orientation, that showed the worst success rate in terms of heart rate detection, and the error was lower than 2 BPM for 64% of the time.

## 6. Conclusions

The presented work demonstrated the ability of an FMCW radar to measure the position and the vital signs (respiratory and heart rate) of a subject in realistic indoor environments. The radar system working at 5.8 GHz and the radiating elements used for the measurements were specifically designed and realized. For the antennas, two identical series-fed arrays, whose patch shape was opportunely studied to reduce the SLL and increase the bandwidth, were proposed to assure a low cost, compact, and well performing system. Thanks to its high gain, the antenna helped to spatially filter the clutter, thus improving the quality of the measurement. Measurements were carried out in a common office environment on five subjects positioned 1.5 m from the radar in the line of sight of the antenna. Four different scenarios were considered: the subject was placed with his/her chest, left side, back, and right side towards the antenna. For one of the subjects, measurements were also repeated at different distances from the radar (from 1 m to 3 m). The signal collected with the FMCW radar was compared with the ones of a respiratory belt and a PPG used as reference gold standards. A statistical analysis was performed on the data collected from the five subjects, revealing that the radar was able to measure both the respiratory and heart rate with elevated accuracy, independently of the chest orientation towards the radar antenna. The maximum error in terms of BPM was 0.8 BPM and 3.1 BPM for the respiration and heart rate, respectively.

## References

[1] Pisa S., Pittella E., Piuzzi E. (2016). A Survey of Radar Systems for Medical Applications. IEEE Aerosp. Electron. Syst. Mag..

[2] Li C., Peng Z., Huang T.Y., Fan T., Wang F.K., Horng T.S., Munoz-Ferreras J.M., Gomez-Garcia R., Ran L., Lin J. (2017). A Review on Recent Progress of Portable Short-Range Noncontact Microwave Radar Systems. IEEE Trans. Microw. Theory Tech..

[3] Gu C. (2016). Short-Range Noncontact Sensors for Healthcare and Other Emerging Applications: A Review. Sensors.

[4] Kebe M., Gadhafi R., Mohammad B., Sanduleanu M., Saleh H., Al-Qutayri M. (2020). Human Vital Signs Detection Methods and Potential Using Radars: A Review. Sensors.

[5] Bernardi P., Cicchetti R., Pisa S., Pittella E., Piuzzi E., Testa O. (2014). Design, Realization, and Test of a UWB Radar Sensor for Breath Activity Monitoring. IEEE Sens. J..

[6] Schleicher B., Nasr I., Trasser A., Schumacher H. (2013). IR-UWB Radar Demonstrator for Ultra-Fine Movement Detection and Vital-Sign Monitoring. IEEE Trans. Microw. Theory Tech..

[7] Lai J.C.Y., Xu Y., Gunawan E., Chua E.C.P., Maskooki A., Guan Y.L., Low K.S., Soh C.B., Poh C.L. (2011). Wireless Sensing of Human Respiratory Parameters by Low-Power Ultrawideband Impulse Radio Radar. IEEE Trans. Instrum. Meas..

[8] Nijsure Y., Tay W.P., Gunawan E., Wen F., Yang Z., Guan Y.L., Chua A.P. (2013). An Impulse Radio Ultrawideband System for Contactless Noninvasive Respiratory Monitoring. IEEE Trans. Biomed. Eng..

[9] Leib M., Schmitt E., Gronau A., Dederer J., Schleicher B., Schumacher H., Menzel W. (2009). A Compact Ultra-Wideband Radar for Medical Applications. Frequenz.

[10] Kim J.D., Lee W.H., Lee Y., Lee H.J., Cha T., Kim S.H., Song K.M., Lim Y.H., Cho S.H., Cho S.H. (2019). Non-Contact Respiration Monitoring Using Impulse Radio Ultrawideband Radar in Neonates. R. Soc. Open Sci..

[11] Shikhsarmast F., Lyu T., Liang X., Zhang H., Gulliver T. (2019). Random-Noise Denoising and Clutter Elimination of Human Respiration Movements Based on an Improved Time Window Selection Algorithm Using Wavelet Transform. Sensors.

[12] Zhang C., Kuhn M., Merkl B., Fathy A., Mahfouz M. (2010). Real-Time Noncoherent UWB Positioning Radar With Millimeter Range Accuracy: Theory and Experiment. IEEE Trans. Microw. Theory Tech..

[13] Lazaro A., Girbau D., Villarino R. (2010). Analysis of vital signs monitoring using an Ir-Uwb Radar. Prog. Electromagn. Res..

[14] Lazaro A., Girbau D., Villarino R., Ramos A. Vital Signs Monitoring Using Impulse Based UWB Signal. Proceedings of the 41st European Microwave Conference.

[15] Xiao Y., Lin J., Boric-Lubecke O., Lubecke V. A Ka-Band Low Power Doppler Radar System for Remote Detection of Cardiopulmonary Motion. Proceedings of the 2005 IEEE Engineering in Medicine and Biology 27th Annual Conference.

[16] Xiao Y., Lin J., Boric-Lubecke O., Lubecke M. (2006). Frequency-Tuning Technique for Remote Detection of Heartbeat and Respiration Using Low-Power Double-Sideband Transmission in the Ka-Band. IEEE Trans. Microw. Theory Tech..

[17] Gu C., Li R., Zhang H., Fung A.Y.C., Torres C., Jiang S.B., Li C. (2012). Accurate Respiration Measurement Using DC-Coupled Continuous-Wave Radar Sensor for Motion-Adaptive Cancer Radiotherapy. IEEE Trans. Biomed. Eng..

[18] Zhang T., Sarrazin J., Valerio G., Istrate D. (2018). Estimation of Human Body Vital Signs Based on 60 GHz Doppler Radar Using a Bound-Constrained Optimization Algorithm. Sensors.

[19] Lubecke O., Ong P.W., Lubecke V. (2002). 10 GHz Doppler Radar Sensing of Respiration and Heart Movement. Proceedings of the IEEE 28th Annual Northeast Bioengineering Conference (IEEE Cat. No.02CH37342).

[20] Gu C., He Y., Zhu J. (2019). Noncontact Vital Sensing With a Miniaturized 2.4 GHz Circularly Polarized Doppler Radar. IEEE Sens. Lett..

[21] Park B.K., Boric-Lubecke O., Lubecke V.M. (2007). Arctangent Demodulation With DC Offset Compensation in Quadrature Doppler Radar Receiver Systems. IEEE Trans. Microw. Theory Tech..

[22] Li C., Lin J. Optimal Carrier Frequency of Non-Contact Vital Sign Detectors. Proceedings of the 2007 IEEE Radio and Wireless Symposium.

[23] Liang Q., Xu L., Bao N., Qi L., Shi J., Yang Y., Yao Y. (2019). Research on Non-Contact Monitoring System for Human Physiological Signal and Body Movement. Biosensors.

[24] Girbau D., Lazaro A., Ramos Á., Villarino R. (2012). Remote Sensing of Vital Signs Using a Doppler Radar and Diversity to Overcome Null Detection. IEEE Sens. J..

[25] Choi C.H., Park J.H., Lee H.N., Yang J.R. (2019). Heartbeat Detection Using a Doppler Radar Sensor Based on the Scaling Function of Wavelet Transform. Microw. Opt. Technol. Lett..

[26] Kim J.Y., Park J.H., Jang S.Y., Yang J.R. (2019). Peak Detection Algorithm for Vital Sign Detection Using Doppler Radar Sensors. Sensors.

[27] Wang G., Gu C., Inoue T., Li C. Hybrid FMCW-Interferometry Radar System in the 5.8 GHz ISM Band for Indoor Precise Position and Motion Detection. Proceedings of the 2013 IEEE MTT-S International Microwave Symposium Digest (MTT).

[28] Wang G., Gu C., Inoue T., Li C. (2014). A Hybrid FMCW-Interferometry Radar for Indoor Precise Positioning and Versatile Life Activity Monitoring. IEEE Trans. Microw. Theory Tech..

[29] Sacco G., Pittella E., Piuzzi E., Pisa S. A Radar System for Indoor Human Localization and Breath Monitoring. Proceedings of the 2018 IEEE International Symposium on Medical Measurements and Applications (MeMeA).

[30] Li H., Shrestha A., Fioranelli F., Le Kernec J., Heidari H. (2019). FMCW Radar and Inertial Sensing Synergy for Assisted Living. J. Eng..

[31] Peng Z., Munoz-Ferreras J.M., Gomez-Garcia R., Li C. FMCW Radar Fall Detection Based on ISAR Processing Utilizing the Properties of RCS, Range, and Doppler. Proceedings of the 2016 IEEE MTT-S International Microwave Symposium (IMS).

[32] Postolache O., Girao P.S., Postolache G., Gabriel J. Cardio-Respiratory and Daily Activity Monitor Based on FMCW Doppler Radar Embedded in a Wheelchair. Proceedings of the 2011 Annual International Conference of the IEEE Engineering in Medicine and Biology Society.

[33] Mercuri M., Lorato I.R., Liu Y.H., Wieringa F., Hoof C.V., Torfs T. (2019). Vital-Sign Monitoring and Spatial Tracking of Multiple People Using a Contactless Radar-Based Sensor. Nat. Electron..

[34] Roussos C. (1995). The Thorax–Part A: Physiology (In Three Parts).

[35] Boric-Lubecke O., Lubecke V.M., Droitcour A.D., Park B.K., Singh A. (2016). Doppler Radar Physiological Sensing.

[36] De Groote A., Wantier M., Cheron G., Estenne M., Paiva M. (1997). Chest Wall Motion during Tidal Breathing. J. Appl. Physiol..

[37] Ramachandran G., Singh M. (1989). Three-Dimensional Reconstruction of Cardiac Displacement Patterns on the Chest Wall during the P, QRS and T-Segments of the ECG by Laser Speckle Inteferometry. Med Biol. Eng. Comput..

[38] Analog Devices HMC587LC4B Data Sheet. https://www.analog.com/media/en/technical-documentation/data-sheets/hmc587.pdf.

[39] Analog Devices (2017). HMC392ALC4 Data Sheet. https://www.analog.com/media/en/technical-documentation/data-sheets/hmc392Alc4.pdf.

[40] Mini-Circuits High Power, DC Pass Power Splitter/Combiner High ZN2PD2-63+. https://www.minicircuits.com/pdfs/ZN2PD2-63+.pdf.

[41] Analog Devices HMC557A Data Sheet. https://www.analog.com/media/en/technical-documentation/data-sheets/hmc557a.pdf.

[42] National Instruments (2015). DEVICE SPECIFICATIONS NI 6361 X Series Data Acquisition: 2 MS/s, 16 AI, 24 DIO, 2 AO. http://www.ni.com/pdf/manuals/374650c.pdf.

[43] Sacco G., D’Atanasio P., Pisa S. (2020). A Wideband and Low-Sidelobe Series-Fed Patch Array at 5.8 GHz for Radar Applications. IEEE Antennas Wirel. Propag. Lett..

[44] Li C., Tofighi M.R., Schreurs D., Horng T.S.J. (2017). Principles and Applications of RF/Microwave in Healthcare and Biosensing.

[45] Yuan T., Yuan N., Li L.-W. (2008). A Novel Series-Fed Taper Antenna Array Design. IEEE Antennas Wirel. Propag. Lett..

[46] Chopra R., Kumar G. (2019). Series-Fed Binomial Microstrip Arrays for Extremely Low Sidelobe Level. IEEE Trans. Antennas Propag..

[47] Lee H., Kim B.H., Park J.K., Yook J.G. (2019). A Novel Vital-Sign Sensing Algorithm for Multiple Subjects Based on 24-GHz FMCW Doppler Radar. Remote Sens..

[48] Anitori L., de Jong A., Nennie F. FMCW Radar for Life-Sign Detection. Proceedings of the 2009 IEEE Radar Conference.

